# ddPCR Overcomes the CRISPR-Cas13a-Based Technique for the Detection of the BRAF p.V600E Mutation in Liquid Biopsies

**DOI:** 10.3390/ijms252010902

**Published:** 2024-10-10

**Authors:** Irina Palacín-Aliana, Noemí García-Romero, Josefa Carrión-Navarro, Pilar Puig-Serra, Raul Torres-Ruiz, Sandra Rodríguez-Perales, David Viñal, Víctor González-Rumayor, Ángel Ayuso-Sacido

**Affiliations:** 1 Atrys Health, 08025 Barcelona, Spain; iripalacin@gmail.com (I.P.-A.); vgrumayor@atryshealth.com (V.G.-R.); 2 Fundación de Investigación HM Hospitales, HM Hospitales, 28015 Madrid, Spain; 3 Faculty of Science, Universidad de Alcalá, 28801 Madrid, Spain; 4 Faculty of Experimental Sciences, Universidad Francisco de Vitoria, 28223 Madrid, Spain; noemi.garcia@ufv.es (N.G.-R.); pepa.carrion@ufv.es (J.C.-N.); 5 Brain Tumor Laboratory, Fundación Vithas, Grupo Hospitales Vithas, 28043 Madrid, Spain; 6 Human Cancer Genetics Program, Centro Nacional de Investigaciones Oncológicas (CNIO), Molecular Cytogenetics & Genome Editing Unit, Melchor Fernández Almagro, 3, 28029 Madrid, Spain; ppuig@cnio.es (P.P.-S.); rtorresr@cnio.es (R.T.-R.); srodriguezp@cnio.es (S.R.-P.); 7 Centro de Investigación Energéticas Medioambientales y Tecnológicas (CIEMAT), Advanced Therapies Unit, Hematopoietic Innovative Therapies Division, Instituto de Investigación Sanitaria Fundación Jimenez Diaz (IIS-FJD, UAM), 28040 Madrid, Spain; 8 Department of Medical Oncology, Hospital Universitario La Paz, 28046 Madrid, Spain; dvinallozano@gmail.com; 9 Faculty of Medicine, Universidad Francisco de Vitoria, 28223 Madrid, Spain

**Keywords:** CRISPR-Cas13a, qPCR, ddPCR, actionable mutations, variant allele frequency, BRAF p.V600E

## Abstract

The isolation of circulating tumoral DNA (ctDNA) present in the bloodstream brings about the opportunity to detect genomic aberrations from the tumor of origin. However, the low amounts of ctDNA present in liquid biopsy samples makes the development of highly sensitive techniques necessary to detect targetable mutations for the diagnosis, prognosis, and monitoring of cancer patients. Here, we employ standard genomic DNA (gDNA) and eight liquid biopsy samples from different cancer patients to examine the newly described CRISPR-Cas13a-based technology in the detection of the BRAF p.V600E actionable point mutation and appraise its diagnostic capacity with two PCR-based techniques: quantitative Real-Time PCR (qPCR) and droplet digital PCR (ddPCR). Regardless of its lower specificity compared to the qPCR and ddPCR techniques, the CRISPR-Cas13a-guided complex was able to detect inputs as low as 10 pM. Even though the PCR-based techniques have similar target limits of detection (LoDs), only the ddPCR achieved a 0.1% variant allele frequency (VAF) detection with elevated reproducibility, thus standing out as the most powerful and suitable tool for clinical diagnosis purposes. Our results also demonstrate how the CRISPR-Cas13a can detect low amounts of the target of interest, but its base-pair specificity failed in the detection of actionable point mutations at a low VAF; therefore, the ddPCR is still the most powerful and suitable technique for these purposes.

## 1. Introduction

Cancer is one of the principal causes of death worldwide [1]. Over the last few decades, many advances have facilitated the identification and verification of new driver mutations that guide clinicians in the diagnosis and prognosis of the different cancer types through the development of more effective treatment selection with novel targeted drugs [2]. BRAF is a serine/threonine kinase in the RAS/RAF/MEK/ERK pathway which, once stimulated, promotes cell growth, survival, and differentiation. BRAF activity can be upregulated by various mechanisms, including mutations [3]. Currently, more than 200 different types of *BRAF* mutations have been identified in various types of cancer, particularly in melanoma, colorectal cancer (CRC), and non-small cell lung cancer (NSCLC) [4,5]. The most relevant and frequent *BRAF* point mutation occurs in exon 15 at codon 600, resulting in an amino acid replacement from valine (V) to glutamic acid (E) named *BRAF* p.V600E [6]. The administration of targeted BRAF inhibitors, such as vemurafenib and dabrafenib, has been shown to improve the response and survival of patients with *BRAF* p.V600E mutations [7].

Although solid biopsies are crucial in cancer diagnosis, they present many limitations such as their invasive obtention methods and the lack of intra-tumoral heterogeneity representation, given that only a small fraction of the tumor is usually obtained [8]. Recently, to overcome these issues, samples obtained from different biofluids such as blood (serum and plasma), saliva, breast milk, cerebrospinal fluid, stool, semen, urine, etc., are emerging [9]. The isolation of the circulating tumoral DNA (ctDNA) released by the tumor arises the opportunity of detecting genomic aberrations [10], giving remarkable information about treatment response, tumoral staging, prognosis, minimal residual disease, and actionable mutations, enhancing more precise clinical decisions [11]. Furthermore, ctDNA has been proven to be a powerful tool since all tumor cells, indistinctly of their phenotype, secrete DNA into the biofluids, providing information of the whole tumor, and revealing a snapshot of the intra-tumoral heterogeneity state at the moment of sample collection [12]. In this context, the development of highly sensitive techniques to detect targetable mutations are crucial for diagnostic, prognostic and monitoring cancer patients. Nowadays the most widely used technologies for biomarkers detection, both in tumor tissue and in liquid biopsy samples, include the quantitative Real-Time PCR (qPCR), Sanger sequencing, Next-Generation Sequencing (NGS) platforms and droplet digital PCR (ddPCR) [13,14]. Thus, even though various methods for *BRAF* mutations are available, they lack crucial clinical needs for rapid, sensitive, and comprehensive detection of actionable mutations.

**Figure 1:**
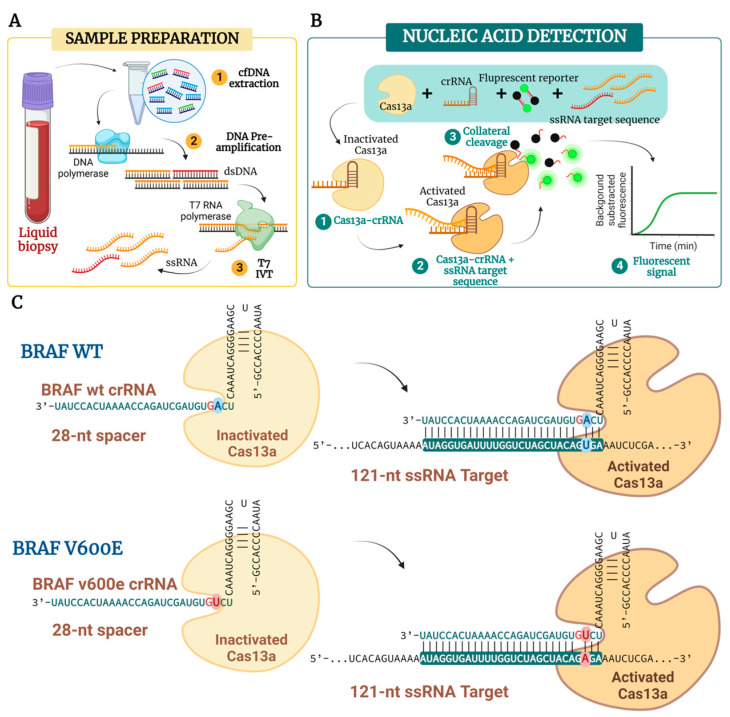
Schematic illustration of nucleic acid mutation detection using CRISPR-Cas13a enzyme collateral cleavage activity. (**A**) Schematic image of sample extraction and steps needed for the ssRNA acquisition. After genomic DNA (gDNA) or circulating free DNA (cfDNA) extraction from the clinical samples, the first step consists of DNA amplification by conventional PCR performed with primers tagged with a T7 promoter sequence. The pre-amplification step will generate double-stranded DNA (dsDNA) amplicons of the target sequence with an appended T7 promoter sequence needed for the next step and for procedure sensibility improvement. Thereafter, the in vitro transcription (IVT) of the PCR product by a T7 polymerase will produce transcribed single-stranded RNA (ssRNA) targets. (**B**) Representation of the collateral cleavage Cas13a: crRNA complex activated by ssRNA target sequence binding. The CRISPR guide sequence (crRNA) contains repeat sequences that will form a loop essential for its anchor to the Cas13a nuclease. Once the Cas13a: crRNA complex has been formed, the crRNA and ssRNA target base-pairing activates the collateral nuclease activity of the Cas13a. This collateral cleavage activity will cleave a fluorescent reporter attached to its quencher by a short ssRNA sequence generating a measurable fluorescent signal. (**C**) Schematic illustration of the Cas13a: crRNA complex. Two different crRNA guide sequences have been designed for the detection of the *BRAF* wild-type (WT) and *BRAF* p.V600E-mutated sequences. The Cas13a: crRNA complex is formed by the loop sequence present in the crRNA. Cas13a is inactive when it is not bound to target ssRNA. Once the complex binds to the ssRNA, the collateral RNAse activity of the Cas13a is initiated. To enhance the single-base pair specificity, an additional synthetic mismatch is placed next to the point mutation of interest (marked in red).

With the aim of creating new powerful and cheaper nucleic acid detection techniques, a new use for the well-known CRISPR-Cas systems as a diagnostic tool has been described [15]. The Cas13, previously known as C2c2, is a relatively new type-VI CRISPR-Cas family member [16]. Recent in vitro studies showed how the Cas13a endonuclease presents collateral RNase activity upon recognition of the target RNA sequence (Figure 1A,B) [17]. This non-specific collateral endonuclease activity has been exploited for the detection of specific RNA sequences by the degradation of fluorescent labeled RNA, lateral flow strips, or gold nanoparticle-based colorimetry [17,18,19].

This new nucleic acid detection platform, combined with an isothermal amplification and T7 in vitro transcription, was described by Feng Zhang and coworkers and named Specific High Sensitivity Enzymatic Reporter UnLOCKing (SHERLOCK) [17,20]. SHERLOCK is a CRISPR-based diagnostic tool that uses custom-designed CRISPR RNA (crRNA) in the guidance of *the Leptotrichia wadei* Cas13a (LwaCas13a) endonuclease for the detection of specific RNA or DNA targets. This SHERLOCK technology promises a femtomolar (fM), or in some applications even attomolar (aM), sensitivity and single-base mismatch specificity [20,21].

The growing interest of clinicians in administering personalized and effective treatments to patients has generated the need to permanently optimize and improve the diagnostic tools available. Therefore, in this study, we firstly aimed to assemble the newly proposed CRISPR-Cas13-based diagnostic tool for the detection of the *BRAF* p.V600E point mutation, as described in Figure 1. Secondly, we compared its sensitivity, specificity, and efficacy with two gold-standard techniques: the qPCR and the ddPCR. As a proof-of-concept study, we used a series of different *BRAF* p.V600E Reference Standard Variant Allele Frequency (VAF) and liquid biopsy samples obtained from a cohort of eight patients diagnosed with CRC and lung adenocarcinoma (LUAD) to test the three nucleic acid detection tools in order to define the most suitable method for DNA biomarker detection. We demonstrate how the CRISPR-Cas13a can detect low amounts of ssRNA target, but its base-pair discrimination specificity needs further optimization. Highly concentrated sample inputs enable the amplification of mutant alleles down to 0.5% using qPCR, but the limit of detection (LoD) deteriorates to 5% as the target concentration decreases. Overall, our data highlight the ddPCR as the most effective DNA biomarker detection tool due to its ability to detect a 0.1% VAF even in low-concentration samples. Furthermore, the ddPCR gives an absolute quantification of the DNA copies per µL of sample and the mutated fractional abundance.

## 2. Results

### 2.1. Cas13a Collateral Cleavage Activity for Nucleic Acid Detection

**Figure 2:**
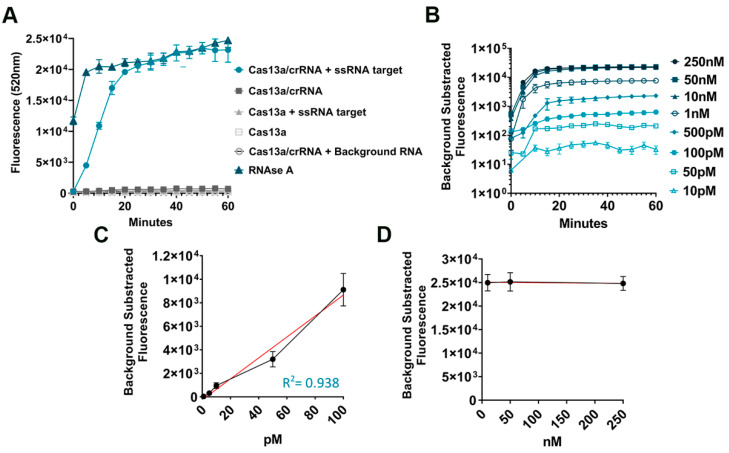
CRISPR-Cas13a ssRNA target detection. (**A**) Fluorescent measurement of Cas13a activity employing 50 nM of ssRNA target. RNase A was used as positive control for the cleavage of the fluorescent RNA reporter. (**B**) CRISPR-Cas13a time-course fluorescence signal intensities expressed in logarithmic scale under different ssRNA target concentration inputs and using the BRAF p.V600E crRNA (10 pM, 50 pM, 100 pM, 500 pM, 1 nM, 10 nM, 50 nM, and 250 nM). Fluorescence measurements were taken every 5 min at 37 °C. (**C**,**D**) Linear relationship between final fluorescent signal (t = 1 h) ((**B**) data) and ssRNA target concentration (10 pM, 50 pM, 100 pM, 500 pM, 1 nM, 10 nM, 50 nM, and 250 nM). *n* = 3 independent experimental reactions with technical duplicates; error bars represent mean ± SD.

For the setup of the CRISPR-Cas13a technology, we wanted to test whether the Cas13a was able to bind to the designed crRNA giving a fluorescent signal. Also, we tested whether the Cas13a could give a false positive signal. For this purpose, we used the BRAF WT crRNA and the ssRNA target amplified from gDNA standards and transcribed to RNA as described in Figure 1A. As shown in Figure 2A, the Cas13a only gives a fluorescent signal in the presence of a crRNA and a complementary ssRNA target. Otherwise, in the presence of only background RNA (isolated from commercial cell lines) with no specific target input, it does not show off any target activity. RNAse A was used as an assay-positive control.

To determine the LoD, serial dilutions of the ssRNA targets were employed. CRISPR-Cas13a exhibits detectable reporter cleavage with as few as 10 pM (equivalent to 4 × 10^−4^ ng/uL, mw = 39,076.98 g/mol) of ssRNA targets with the BRAF p.V600E crRNA designed (Figure 2B) and 50 pM with the BRAF WT crRNA (Supplementary Figure S1A). A significantly detectable fluorescent signal was obtained in 15 min after mixing the CRISPR-Cas13 with the target RNA, and then, it reached a *plateau*.

In the LoD assay, an association between the ssRNA target concentration and the fluorescent signal was detected. When plotting the final fluorescence signals against the ssRNA target concentrations from 10 pM to 100 pM, a linear relationship (R^2^ = 0.938) was observed (Figure 2C). However, the fluorescence signal becomes saturated and reaches a *plateau* at higher concentrations (10, 50, and 250 nM) (Figure 2D).

### 2.2. CRISPR-Cas13a:crRNA Complex Detects Low BRAF p.V600E Allele Frequencies

We followed the CRISPR-Cas13a complex characterization by evaluating its sensitivity and specificity. Considering that the VAF present in blood samples tends to be very low [22], the *BRAF* p.V600E standard gDNA with an allelic frequency of 50% was diluted using the corresponding WT standard to generate a wide range of *BRAF* p.V600E VAFs going from 50% to as low as 0.1%.

Using the BRAF p.V600E crRNA, we attempted to see whether the Cas13a could discriminate 0.1% of mutations over 99.9% of WT sequences. This specificity was tested in different transcribed ssRNA target concentrations, from 250 nM to 100 pM.

**Figure 3:**
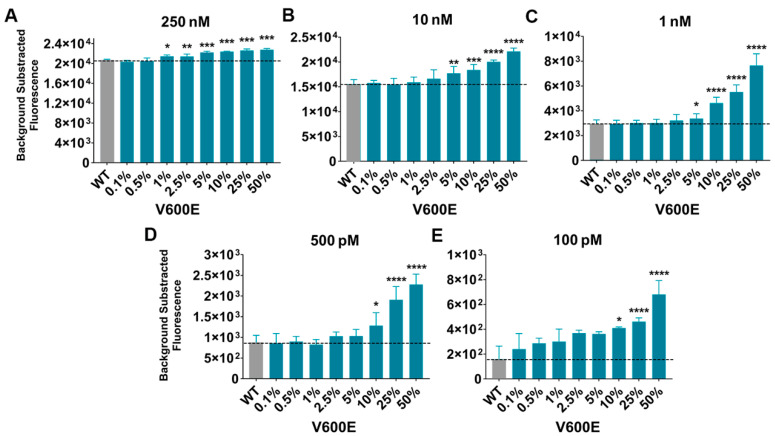
CRISPR-Cas13a *BRAF* p.V600E mutation detection. CRISPR-Cas13a minor allele frequency detection using different ssRNA target inputs: (**A**) 250 nM, (**B**) 10 nM, (**C**) 1 nM, (**D**) 500 pM, (**E**) 100 pM. *n* = 3 independent experimental duplicates; all readings were made after 1h of reaction incubation; bars represent mean ± SD; two-tailed *t* test against the WT ssRNA (grey): *, *p* < 0.05; **, *p* < 0.01; ***, *p* < 0.001; ****, *p* < 0.0001.

The results revealed an elevated fluorescent signal in all ssRNA targets at a concentration of 250 nM. Even though, a correlative signal tendency with the mutant allele frequency is noticeable, the Cas13a gave an elevated fluorescent emission in all targets even in the WT sample (Figure 3A). Unfortunately, we found that the technique presented non-specific fluorescent signal when using a WT ssRNA target with the BRAF p.V600E crRNA guide (Figure 3). The BRAF WT crRNA assays are in Supplementary Figure S2.

The conditions with less unspecific signals turned out to be the ssRNA target at 10 nM, 1 nM, 500 pM, and 100 pM (Figure 3 and Figure S1B). Despite this unspecific signal, in all the tests, the CRISPR-Cas13a:crRNA complex could detect VAFs of 10% (Figure 3E), 5% (Figure 3C,D), and even 1% (Figure 3A).

### 2.3. qPCR Detects 0.5% BRAF p.V600E VAF

The proposed new diagnostic tool based on the CRISPR-Cas13a complex was compared with qPCR sensitivity and specificity. This technique is widely used as a diagnostic method to detect the WT and mutant alleles separately using different probes. The technique is able to detect until at least a VAF of 5% [23]. The same range of mutated allele frequencies employed in the CRISPR-Cas13a assays were tested.

**Figure 4:**
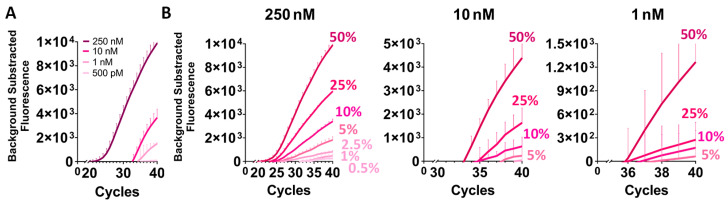
qPCR *BRAF* p.V600E limit of detection characterization. CRISPR-Cas13a minor allele frequency detection using different ssRNA target inputs. (**A**) qPCR *BRAF* p.V600E signal amplifications under different inputs of target concentrations (250 to 1 nM). (**B**) qPCR mutant allele frequency. *n* = 3 independent experimental assays; bars represent mean ± SD; two-tailed *t* test.

The qPCR was able to amplify targets until 1 nM of DNA target concentration, which was its achieved LoD (Figure 4A and Supplementary Figure S3A). At 500 pM, the technique could barely amplify 50% VAF (Figure 4A and Supplementary Figure S3B). The qPCR could amplify a mutant allele frequency of 0.5% when the target concentration was 250 nM. Reducing the input concentration, the minimum VAF detectable changed to 5% for the 10 and 1 nM concentrations (Figure 4B and Supplementary Figure S3C). The qPCR *BRAF* WT detection data are in Supplementary Figure S3B,D. Unfortunately, the qPCR could not amplify DNA targets at 100 pM.

### 2.4. ddPCR BRAF p.V600E VAF Absolute Quantification

**Figure 5:**
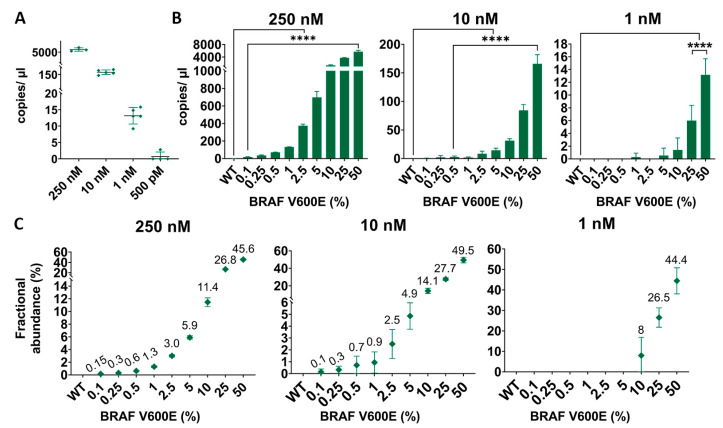
ddPCR *BRAF* p.V600E limit of detection characterization. CRISPR-Cas13a minor allele frequency detection using different ssRNA target inputs. (**A**) ddPCR *BRAF* p.V600E input quantification under different inputs of target concentrations (250 to 1 nM). (**B**) ddPCR mutant allele frequency detection. (**C**) Sample fractional abundance. *n* = 3 independent experimental assays; bars represent mean ± SD; two-tailed *t* test; ****, *p* < 0.0001.

ddPCR is a PCR-based method that provides a precise and highly sensitive absolute quantification of the sample [24]. The ddPCR target concentration LoD was similar to the qPCR LoD, 1 nM, which is approximately 25 copies/µL (0.04 ng/µL) of DNA target concentration (Figure 5A and Supplementary Figure S4A). However, the ddPCR was sensitive enough to detect and quantify the lowest VAF, 0.1% (Figure 5B and Supplementary Figure S5). When using 1 nM of input, the ddPCR loses sensibility and detects until 10% of mutations, and at 500 pM, approximately 4 copies/µL (0.02 ng/µL), it can detect 25 and 50% of mutant alleles, although the signal becomes inconsistent and not entirely reliable (Supplementary Figure S4B,C and Supplementary Table S3). 

Apart from absolute copies/µL quantification of the sample, one of the noteworthy features of the ddPCR is its ability to estimate the proportion of mutant alleles present in the sample, denominated as the fractional abundance (FA). In our assays, the ddPCR was able to accurately calculate the FA of the samples (Figure 5C). In low-concentration samples (1 nM and 500 pM), it only estimated those with higher VAFs—50%, 25%, and 10%—but with less precision (Supplementary Figure S4D).

### 2.5. ddPCR Presents Elevated Experiment Reproducibility

**Figure 6:**
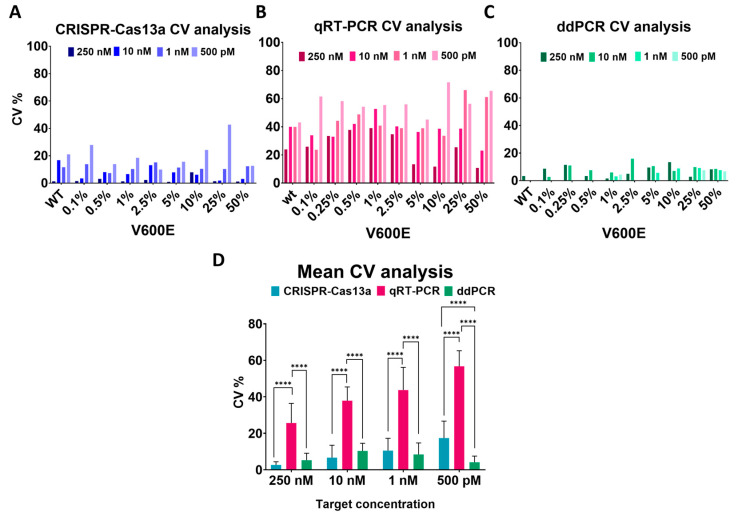
Comparison of CRISPR-Cas13a reproducibility to other nucleic acid detection tools. (**A**) Coefficient of variation of CRISPR-Cas13a minor allele frequency detection using different ssRNA target inputs. (**B**) Coefficient of variation of qPCR minor allele frequency detection using different target inputs. (**C**) Coefficient of variation of ddPCR minor allele frequency detection using different target inputs. (**D**) Mean coefficient of variation for different target inputs and the three detection methods. *n* = 3 independent experimental duplicates; bars represent mean ± SD; two-tailed *t* test: ****, *p* < 0.0001.

The reproducibility of the techniques assayed in this manuscript was evaluated through an analysis of the coefficient of variation (CV). For this purpose, all the data obtained from the experimental replicates of each test, technique, and concentration variations were used (Figure 6).

The CRISPR-Cas13a technology with ssRNA targets from 250 nM to 500 pM presented a CV lower than 20%. The data reading became more inconsistent when increasing the CV to 52 and even 65% when employing ssRNA *BRAF* 0.1% VAF and *BRAF* WT at 100 pM (Figure 6A). In the qPCR experimental replicates, most of the data presented an elevated CV, but it was more pronounced at a 500 pM target concentration (Figure 6B). Lastly, the ddPCR assays presented in all the cases a low CV even in the low-concentration samples (Figure 6C).

Calculating the mean CV of all VAFs per ssRNA concentration, we could see how the CRISPR-Cas13a and the ddPCR techniques present similar reproducibility rates. Both techniques with ssRNA concentrations from 250 to 1 nM do not exceed the 10% CV. However, in the ssRNA targets at 500 pM, the CRISPR-Cas13a presents a higher CV of 17%, and in contrast, the ddPCR at 4%. In summary, these techniques presented less variation than the routinely used qPCR, for which its lowest and highest CVs are 25 and 56%, respectively (Figure 6D).

### 2.6. Detection of BRAF p.V600E Mutation in cfDNA from Different Cancer Types

The techniques were further applied for the detection of the *BRAF* p.V600E mutation in cfDNA purified from plasma samples. As we show in Table 1, for the main purpose of comparing the three techniques, we selected a small pilot cohort of a total of eight LUAD and CRC samples for this analysis due to their high population prevalence but, more importantly, because both tumor types can harbor the *BRAF* p.V600E mutation.

**Figure 7:**
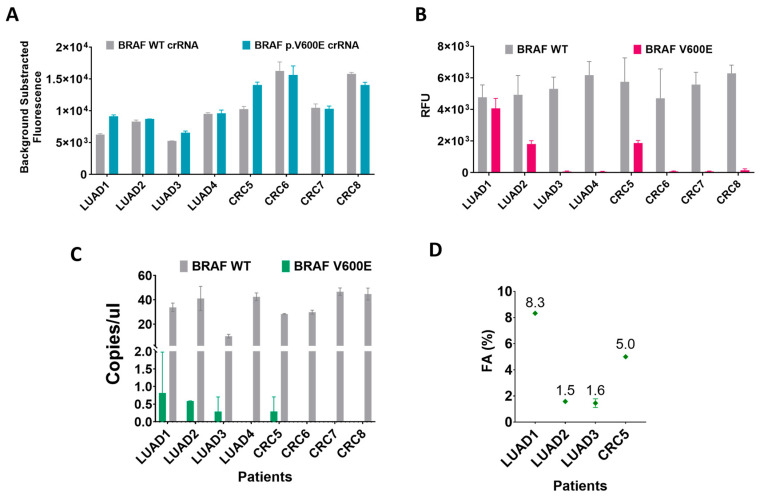
Detection of *BRAF* p.V600E from lung and colorectal cancer patients’ cfDNA by CRISPR-Cas13a, qPCR, and ddPCR. (**A**) CRISPR-Cas13a collateral activity for the detection of *BRAF* p.V600E alteration. (**B**) qPCR *BRAF* p.V600E alteration amplification employing a WT and altered probes. (**C**) ddPCR *BRAF* p.V600E alteration quantification employing a WT and altered probes. (**D**) Sample fractional abundance identified via ddPCR. *n* = 2 independent experimental duplicates.

Among the eight patients’ tissue samples, three of them contained the *BRAF* p.V600E mutation, four were genotyped as *BRAF* WT and one unknown. For this assay, all sample concentrations were adjusted to 5 ng/µL. Using the CRISPR-Cas13a complex and the cfDNA amplified and transcribed to ssRNA, we obtained positive fluorescent signals working with the WT and V600E crRNA guides, but we could not establish with certainty the presence of the *BRAF* p.V600E mutation in the samples (Figure 7A). The lack of a proper WT ssRNA control sample makes establishing a signal threshold to determine which sample is giving a *BRAF* p.V600E positive fluorescence difficult.

With the qPCR, we were able to detect the mutation in two of the three samples genotyped as *BRAF*-positive. The qPCR was unable to detect the *BRAF* p.V600E mutation in the LUAD3 sample but reported a positive signal in the sample CRC5, from which we do not know the *BRAF* status in the tissue samples (Figure 7B).

In contrast to the other techniques, the ddPCR detected the *BRAF* mutation in all the positive samples (LUAD1, LUAD2, and LUAD3), and as previously reported by qPCR, the ddPCR also detected mutation in the CRC5 sample (Figure 7C). Beyond that, using the *BRAF* WT absolute quantification as a reference, the ddPCR calculated the mutation fractional abundance present in the *BRAF*-positive samples, with the LUAD1 being the one that presented more mutations (Figure 7D).

A comprehensive summary of the results is presented in Table 2, encapsulating the key findings and providing a consolidated overview of the data discussed throughout this manuscript.

## 3. Discussion

The emergence of novel Cas endonucleases, such as Cas13a [25], presents novel approaches for targeted nucleic acid sequence detection, enabling the identification of point mutations, deletions, insertions, and others [15,26,27,28]. Since its discovery, different researchers have adapted this technology due to their interest in the detection of micro-RNAs (miRNAs) [29,30,31,32], mitochondrial mutations [33], pathogenic bacteria [34,35,36], species for ecological studies [37], and even hepatitis B virus mutations [38]. However, despite the myriad of applications enabled by this technology, the assessment and comparison of its efficacy in identifying point mutations have not been thoroughly conducted against other established and routinely employed methods. Accordingly, to find an optimal method for routine clinical diagnosis, in this study, the CRISPR-Cas13a technology has been optimized and compared with two habitually used methods, qPCR and ddPCR, for the detection of the *BRAF* mutation p.V600E.

In our optimization assays, the crRNA-guided CRISPR-Cas13a demonstrated its target specificity since its collateral cleavage only activates upon target match and did not generate false positive signals with the negative control containing background RNA. However, in our assays, we could only detect target inputs as low as 10 pM, and not the fM LoD achieved by other publications [20,21]. We were not able to reach this level of sensitivity probably due to the samples employed since we did not employ synthesized DNA samples to mimic a real-life sample. Furthermore, we used different ssRNA IVT transcription enzymes and a different pre-amplification step prior to the Cas13a detection. The collateral cleavage activity is able to generate a fluorescent signal within 15–20 min after reaction initiation, which is approximately twice as rapid as any PCR-based assays. Furthermore, fluorescence measurements from Cas13a detection are correlated with input RNA concentration. Other researchers have demonstrated that with further optimization, the detection with Cas13a can even be quantitative [18,20].

Since the *BRAF* p.V600E mutation present in tumor samples can oscillate in the range from 50% to less than 0.1% [22,39,40], a diagnostic tool needs to be able to properly detect the lower mutation frequencies. Hence, we made a series of VAF samples to visualize the technique LoD. The results revealed unspecific signals due to the elevated fluorescence emitted when using a completely WT ssRNA target with the mutated guide crRNA BRAF p.V600E. Our findings are consistent with those of other publications where they also found CRISPR-Cas13a unspecific signals [20,33]. This elevated false positive signal may be caused by the saturation of the Cas endonuclease which binds to the WT or mutated ssRNA, indistinctly raising the positive readings. Thus, the technique needs further optimization to properly discriminate its target by one base pair. Our results show that this method has shown high sensitivity for the detection and identification of the BRAF sequence even with target concentrations of 100 pM or a VAF of 5%. Unfortunately, the CRISPR-Cas13a nonspecific signal makes the incorporation of a control mandatory to be able to establish a threshold to discriminate the positive signals from the negative ones.

By comparing the CRISPR-based mutation detection method with the conventional techniques, we found that the Cas13a endonuclease can detect lower amounts of input than the PCR-based techniques. However, when it comes to the VAF LoD, the Cas13a and the qPCR can similarly detect until 5% of mutations. So, these results do not improve the VAF LoD sensitivity and specificity already achieved by the implemented diagnostic tools in the clinic [23,40,41].

The ddPCR has presented many diagnosis advantages since its appearance. In our study, even though the ddPCR input LoD was equal to that of the qPCR, we could see how its droplet-separated PCR reaction permitted the absolute quantification of the copies of target per µL of sample and significantly detected a VAF as low as 0.1%. Furthermore, this technique makes it possible to calculate the fractional abundance of the exact mutated allele present in the sample. Moreover, the ddPCR presents a high reproducibility rate measured by less than 10% of the CV across the experimental replicates. The CRISPR-Cas13a and qPCR techniques did not perform as well on the reproducibility test, as they exceeded 10% of the CV.

Due to its powerful technology, the ddPCR has gained much awareness for the detection of genetic cancer aberrations present at low levels in the bloodstream. The technique is further being used not only for point mutation detection and absolute quantification but also for copy number variation assays [42], DNA methylation, and gene rearrangements screening in different sources of clinical samples but principally on plasma, serum, or CSF samples [10]. Researchers are mainly employing the ddPCR for disease management either by genotyping the tumor for diagnostic purposes, for monitoring the tumor status, or for the early detection of drug resistance or sensitivity-associated mutations before and during treatment in patients [11,24,41,43]. The main aim of this study was to evaluate the best technique for properly detecting the *BRAF* p.V600E mutation in ctDNA released into the bloodstream. A small pilot cohort of eight plasma samples from patients were used for technique comparison. Among those samples, three were tissue genotyped as *BRAF* p.V600E and four as *BRAF* WT. The *BRAF* status in the CRC5 sample was unknown since the mutational status of that patient’s tumor was not studied by the hospital. Using the CRISPR-Cas13a for the *BRAF* p.*V600E* point mutation detection was unsuccessful. The unspecific fluorescent signal and the lack of an appropriate background noise control made it impossible to discriminate between positive and false positive signals. In liquid biopsy samples, the incorporation of a representative or appropriate control is quite difficult since the concentration and integrity of the ctDNA fraction is very variable among patients and even in samples acquired from the same patient [44]. Furthermore, the VAF present in such a source of samples tends to oscillate between 5% or even 0.1% [22,39,40]. The Cas13a technique requires further optimization in this regard prior to clinical practice application. The PCR-based techniques could detect the mutations in almost all the samples. However, the qPCR was unable to amplify the LUAD3 *BRAF* mutation probably due to the cfDNA’s low quality and quantity. In this regard, the ddPCR exceeded expectations as a detection tool since it detected the mutation in all the positive samples and confirmed the presence of the *BRAF* p.V600E mutation in the CRC5 samples previously reported by qPCR. We demonstrated that the study of the mutational profile of a tumor via the cfDNA present in a blood sample can guide clinicians to a personalized and precise treatment for cancer patients. Now, the CRC5 patient may benefit from anti-BRAF therapy. The powerful capacity of mutation detection reported by our assay and by many other researchers [11] makes the ddPCR the better technique for liquid biopsy applications.

One of the main challenges of liquid biopsy utility in clinical practice is ensuring a high accuracy for biomarker detection. Among the technologies assayed in this manuscript, the ddPCR possesses robust clinical sensitivity, specificity, and reproducibility for the profiling of actionable mutations present in the ctDNA released in the bloodstream by the tumor of origin. Thus, the use of ddPCR will significantly improve patients’ diagnosis and treatment administration.

## 4. Materials and Methods

### 4.1. Human Samples

Liquid biopsies were obtained from 8 patients diagnosed with LUAD or CRC (Table 1). The LUAD blood samples were obtained from Andalusia’s Public Health System biobank (Spain) (PT17/0015/0041). The colorectal cancer blood samples were collected from donors from La Paz Hospital (PT17/0015/0025) (Madrid, Spain). Permission for their use was obtained from the ethical review board in HM Hospitals (CEIm Nº 21.03.1809-GHM) (Madrid, Spain) and La Paz Hospital (CEIm Nº PI-5062). The LUAD blood samples were included based on the following criteria: all patients had an anatomical pathology confirmation of the presence of any actionable mutation, and there was sufficient cfDNA available. The exclusion criterion was set for samples provided from patients younger than 18 years old. For the CRC blood samples, the inclusion criteria comprised patients with histologically confirmed advanced colorectal cancer, specifically at a stage higher than stage 2, and there was enough of the sample. The exclusion criterion was applied to patients already undergoing any form of therapy and younger than 18 years old. The Pathological Anatomy Service provided tissue molecular profiles of each sample assessed by NGS or the therascreen *BRAF V600E* RGQ PCR kit (Qiagen, Barcelona, Spain). All patients provided written informed consent before enrollment. All research procedures conformed to the principles of the Helsinki Declaration. Plasma was obtained from peripheral blood samples by centrifugation and stored at −80 °C until cfDNA isolation.

### 4.2. Circulating Tumor DNA Isolation and Quantification

For plasma obtention, whole blood collected into EDTA-treated tubes was centrifuged at 1600× *g* for 10 min. Plasma was collected and centrifugated again at 3200 g for 10 min and finally, stored at −80 °C until use. Total circulating DNA was extracted from 1 mL of plasma with the QIAmp Circulating Nucleic Acid Kit (Qiagen, Madrid, Spain) following the manufacturer’s protocol. Then, DNA concentration was determined using the SimpliNano Spectrophotometer (Biochrom, Madrid, Spain).

### 4.3. Cas13a crRNA and ssRNA Target Preparation

For the CRISPR technique setup, gDNA standards simulating actual patient ctDNA samples were purchased from the commercial vendor Horizon Discovery Group (Cambridge, UK). The *BRAF* WT (HD249) and 50% *BRAF* p.V600E (HD238) Reference Standards were mixed properly to create the 25 to 0.1% *BRAF* p.V600E series.

The gDNA was previously amplified with a conventional PCR with the Paq5000 DNA polymerase kit (Agilent Technologies Madrid, Spain), and primers were ordered from IDT (Integrated DNA technologies) (Supplementary Table S1).

The forward primer contained the T7 promoter sequence, 5′-GAAATTAATACGACTCACTATAGGG-3′, for later conversion of DNA to RNA. The amplified gDNA of the region of interest with the appended T7 promoter sequence was incubated with a T7 RNA polymerase overnight at 30 °C using the HyperScribe™ T7 High Yield RNA Synthesis Kit (APExBio Technology LLC, Houston, TX, USA) (Figure 1B). For DNA cleanup, an RNase-free DNase (Promega Biotech Iberica SL, Madrid, Spain,) treatment was performed. ssRNA integrity was assessed via an 2% agarose gel and RNA-stained via GelRed Nucleic Acid Gel Stain (Biotium, Fremont, CA, USA).

For the LoD assay, serial dilutions of the targets BRAF WT and BRAF p.V600E ssRNA were made to achieve a series of different target concentrations: 250 nM, 50 nM, 10 nM, 1 nM, 500 pM, 100 pM, 50 pM, and 10 pM.

The crRNAs used and specified in Supplementary Table S2 were ordered directly as RNA from the IDT CRISPR Custom Guide RNAs service.

### 4.4. LwaCas13a Protein

The LwaCas13a protein was provided by Integrated DNA technologies (IDT, Madrid, Spain) at a concentration of 72 µM and stored at −80 °C in small aliquots until use.

### 4.5. Cas13a Collateral Cleavage Assay

Each reaction was performed using a 20 µL total volume and contained 45 nM LwCas13a, 45 nM crRNA, 125 nM RNase Alert fluorescent reporter (IDT, Madrid, Spain), 20–40 ng background RNA, and varying amounts of nucleic acid target indicated in each assay. The reaction was performed in a nuclease assay buffer containing 50 mM NaCl, 10 mM Tris-HCl, 10 mM MgCl_2_, and 20 mM HEPES, pH 8. The detection reactions were conducted in Falcon^®^ 384-well Optilux Black Flat Bottom plates (Corning Inc, Corning, NY, USA) and incubated for 1 h at 37 °C on a Varioskan Flash plate reader (Thermo FisherScientific, Madrid, Spain) with fluorescence measurements taken every 5–10 min. A positive signal was considered when it was significantly higher than the negative control. The collateral cleavage assay was performed with three independent experimental replicates with technical duplicates.

The background RNA was isolated from VERO (RRID: CVCL_0059) and QM7 (RRID: CVCL_3450) commercial cell lines kindly gifted by Dr. Estanislao Nistal. All cells were periodically authenticated by morphologic inspection in the past 3 years. All RNA isolation was performed with mycoplasma-free cells, tested with the Mycoplasma PCR Detection Kit (Applied Biological Materials, Richmond, BC, Canada).

### 4.6. Quantitative Real-Time PCR (qPCR)

For the qPCR mutation detection, the Quantimix easy probes kit (Biotools, Madrid, Spain)with the Thermo Fisher Scientific *BRAF* p.V600E probe was used. The reactions were carried out in a CFX Connect Real-Time PCR Detection System (Bio-Rad, Madrid, Spain). The normalized expression and raw florescent readings were processed via the CFX Manager (v.3.1). A positive signal was considered when the fluorescence intensity surpassed the threshold set. Three independent experimental assays with technical duplicates were performed.

### 4.7. Digital Droplet PCR (ddPCR) Quantification

For DNA target quantification, the ddPCR Supermix for Probes (no dUTP) (Bio-Rad, Madrid, Spain with the Thermo Fisher Scientific *BRAF* P.V600E probe were used. Droplets were performed according to manufacturer’s instructions using the QX200™ Droplet Generator, transferred to a PCR plate, and sealed with a foil heat seal (Bio-Rad, Madrid, Spain) and the PX1™ PCR Plate Sealer (Bio-Rad, SpainPCR amplification was performed on the Bio-Rad C1000 Touch^TM^ thermal cycler (Bio-Rad, Madrid, Spain)as described in the manufacturer’s protocol, and the nucleic acid concentrations were determined via measurement on a QX200 droplet reader. The absolute quantity of DNA (copies/μL) was processed using the QX Manager Standard Edition Software (v 1.2.345, Bio-Rad, Madrid, Spain). Positive droplets were defined based on fluorescence amplitude significantly above the background signal of negative droplets and if the total DNA concentration was greater than 1 copy/µL in at least one replicate. Three separate experimental assays were conducted, each with technical duplicates.

The data were analyzed also using the QX Manager Software (v 1.2.345, Bio-Rad, Madrid, Spain). The absolute quantification mode was used for all ddPCR measurements, and the sample fractional abundance (FA) was calculated as follows:

FA = absolute quantification of mutant clone/(absolute quantification of mutant + wild-type clones)

### 4.8. Coefficient of Variation (CV)

The coefficient of variation defined as the ratio of the standard deviation (σ) to the absolute value of the mean (|xᒣ) was calculated using the descriptive statistics tool of GraphPad Prism, v 9.0.2, as follows:

CV = (σ/|xᒣ) × 100

### 4.9. Statistical Analysis

Statistical analyses were performed in GraphPad Prism, v 9.0.2. The experimental results were statistically analyzed using a t-test for continuous variables and ANOVA. The obtained *p* values were adjusted using Tukey’s method. The family-wise alpha threshold and confidence level of α = 0.05 were used for hypothesis testing as statistically significant levels. The data in the graphs are presented as mean ± SD. * *p* ≤ 0.05; ** *p* ≤ 0.01; *** *p* ≤ 0.001; and **** *p* ≤ 0.0001.

## Figures and Tables

**Table 1:** Features of the patients’ liquid biopsies.

Patients Nº	ID	Cancer Type	Tissue Genotyping	cfDNA (ng/µL)
1	LUAD1	LUAD	*BRAF* p.V600E	12.7
2	LUAD2	LUAD	*BRAF* p.V600E	14.8
3	LUAD3	LUAD	*BRAF* p.V600E	7.7
4	LUAD4	LUAD	*BRAF* WT	27.2
5	CRC5	CRC	Unknown	21.7
6	CRC6	CRC	*BRAF* WT	26.6
7	CRC7	CRC	*BRAF* WT	14.5
8	CRC8	CRC	*BRAF* WT	19

LUAD = lung adenocarcinoma; CRC = colorectal cancer; WT = wild type.

**Table 2:** Comprehensive data summary of the CRISPR-Cas13a, qPCR, and ddPCR techniques.

Parameters Tested	CRISPR-Cas13a	qPCR	ddPCR
Sample type	ssRNA	DNA	DNA
Time of sample obtention	4 h	4 h	4 h
Sample pre-processing	12 h	-	-
Time for results	15 min	1–2 h	3–4 h
Limit of target detection	10 pM	500 pM	500 pM
Limit of mutation detection	1% at 250 nM	0.5% at 250 nM	0.1% at 250 nM
	5% at 10–1 nM	5% at 10–1 nM	0.5% at 10 nM
	10% at 500–100 pM	-	25% at 1 nM
Reproducibility	CV < 20%	CV 25 to 56%	CV < 5%
Number of samples	384 well plate	384/ 96 well plate	96 well plate
“Plexing” of the assay	No	Duplex	Duplex
Mutation detection in patient’s liquid biopsies	No	Yes	Yes

CV, coefficient of variance.

## Data Availability

The data generated and analyzed during this study are included in this published article and its Supplementary Materials.

## Supplementary Materials

The following supporting information can be downloaded at https://www.mdpi.com/article/10.3390/ijms252010902/s1.
