# Supplementary material for: ddPCR Overcomes the CRISPR-Cas13a-Based Technique for the Detection of the BRAF p.V600E Mutation in Liquid Biopsies
Source: Int J Mol Sci. 2024 Oct 10;25(20):10902. doi: 10.3390/ijms252010902 (PMC11507125; doi:10.3390/ijms252010902)
Supplement: Supplementary file 1 [file ijms-25-10902-s001.zip › ijms-3221029-supplementary.pdf]

International Journal of
Molecular Sciences

MDPI

Supplementary Figure legends

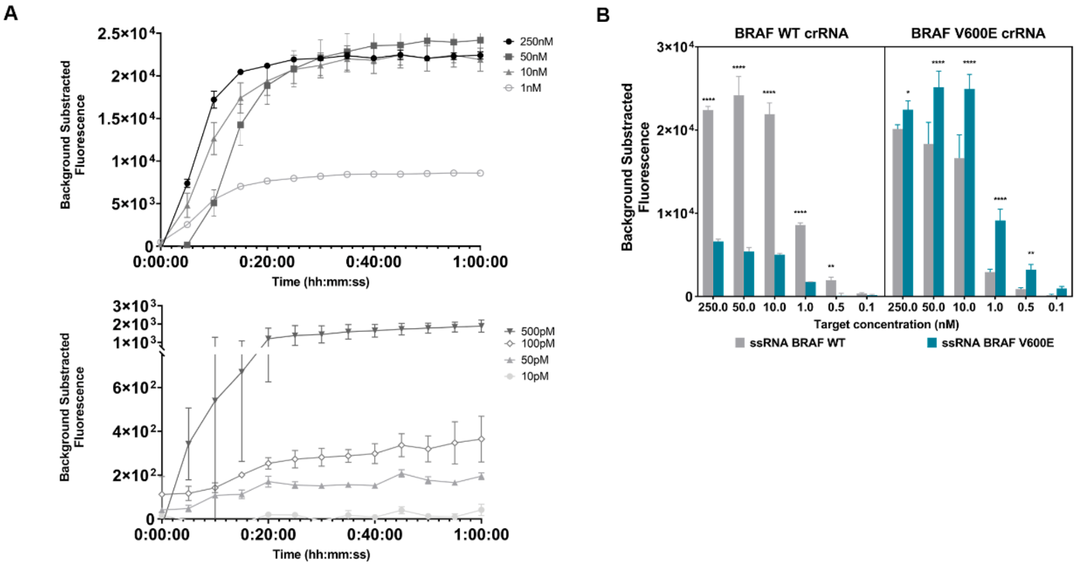

Supplementary Figure S1. CRISPR-Cas13a ssRNA target detection. (A) CRISPR-Cas13a time-course fluorescence signal intensities under different ssRNA target concentration inputs using the BRAF WT crRNA (10 pM, 50 pM, 100 pM, 500 pM, 1nM, 10 nM, 50 nM, and 250 nM). Fluorescence measurements were taken every 5 minutes at 37ºC. **(B)** CRISPR-Cas13a BRAF WT and BRAF 100% p.V600E mutation detection employing different ssRNA target concentrations (250 nM, 50 nM, 10 nM, 1 nM, 500 pM, and 100 pM).

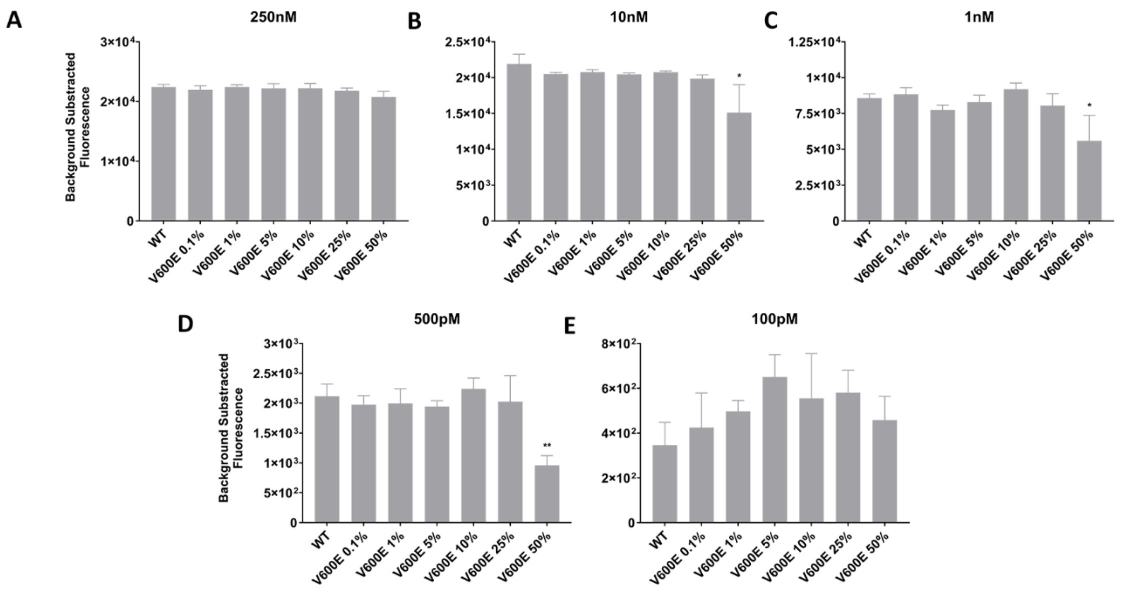

Supplementary Figure S2. CRISPR-Cas13a BRAF WT ssRNA detection. CRISPR-Cas13a BRAF WT crRNA employed for the detection of the BRAF WT ssRNA target at different concentration inputs: (**A)** 250 nM, **B** 10nM, (**C)** 1 nM, (**D)** 500 pM, and (e) 100 pM. n= 3 independent experimental duplicates; bars represent mean ± SD; two-tailed t test: *, p< 0.05; **, p<0.01; ***, p<0.001; ****, p<0.0001.

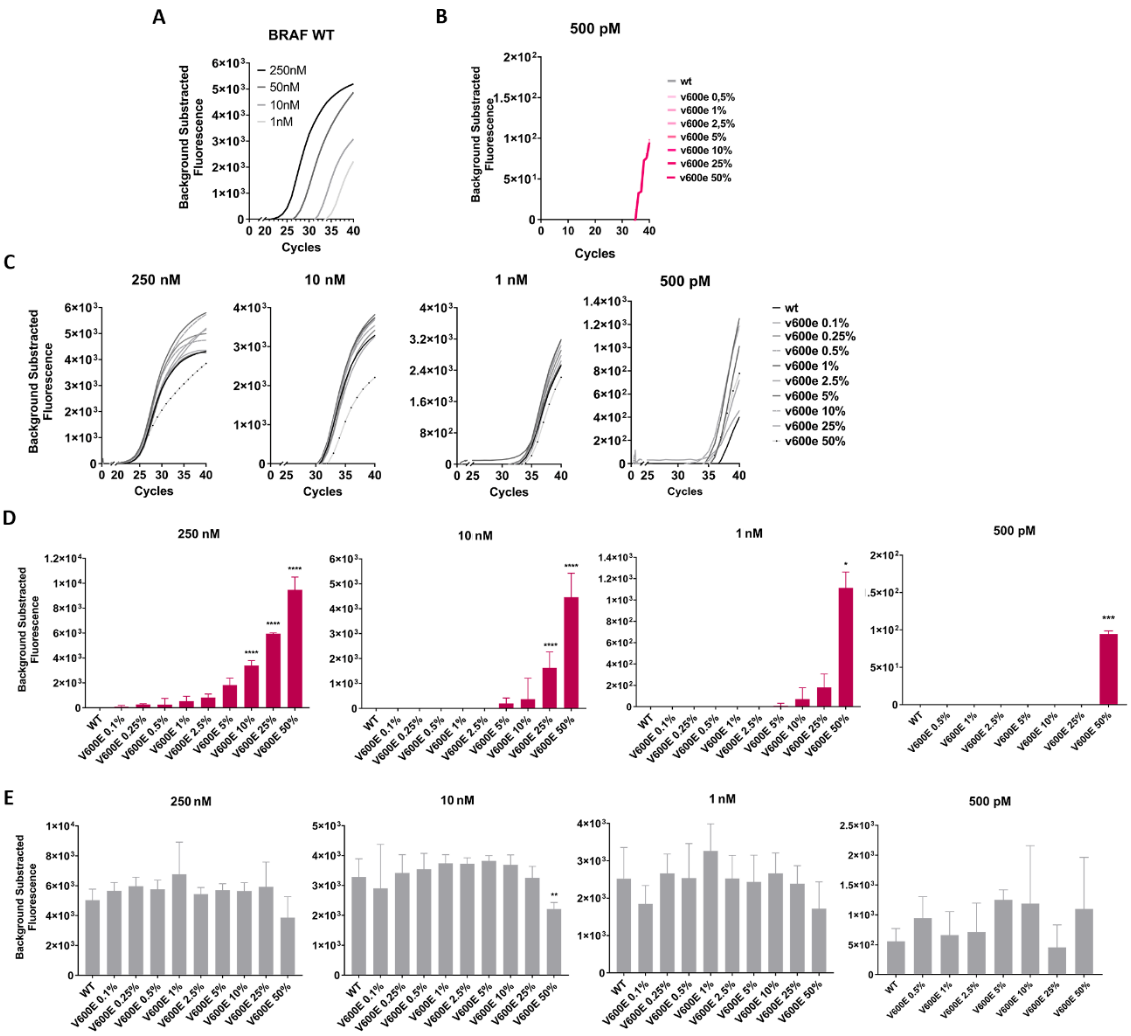

Supplementary Figure S3. qRT-PCR BRAF p.V600E limit of detection characterization and BRAF WT allele assays. **(A)** qRT-PCR BRAF WT signal amplifications under different inputs of target concentrations (250 to 1 nM). **(B)** qRT-PCR mutant allele frequency at 500 pM. **(C)** qRT-PCR BRAF WT amplification of the different VAF cohorts employed and different DNA concentration inputs (250 to 500 pM). **(D)** qRT-PCR bar graph of the mutant allele frequency of Figure 4B. **(E)** qRT-PCR bar graph of the BRAF WT fraction of Supplementary Figure S3B.

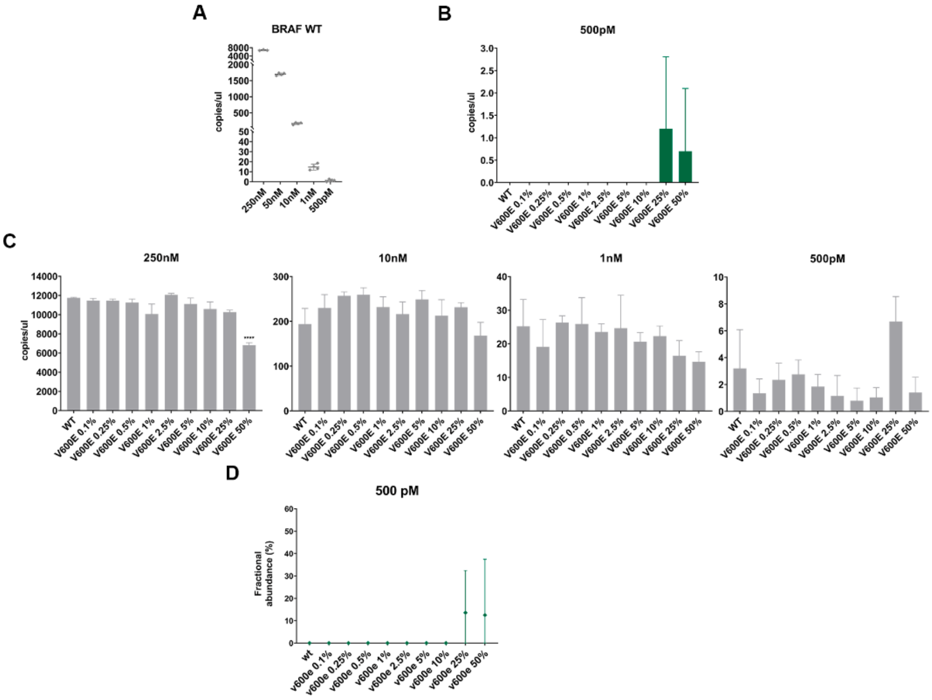

Supplementary Figure S4. ddPCR BRAF p.V600E limit of detection characterization and BRAF WT allele assays. (A) ddPCR BRAF WT input quantification under different inputs of target concentrations (250 to 500 pM). **(B)** ddPCR mutant allele frequency detection with an input DNA concentration at 500pM. **(C)** ddPCR BRAF WT allele of the different VAF cohorts employed and different DNA concentration inputs (250 to 500 pM). **(D)** Sample fractional abundance at a DNA concentration of 500 pM.

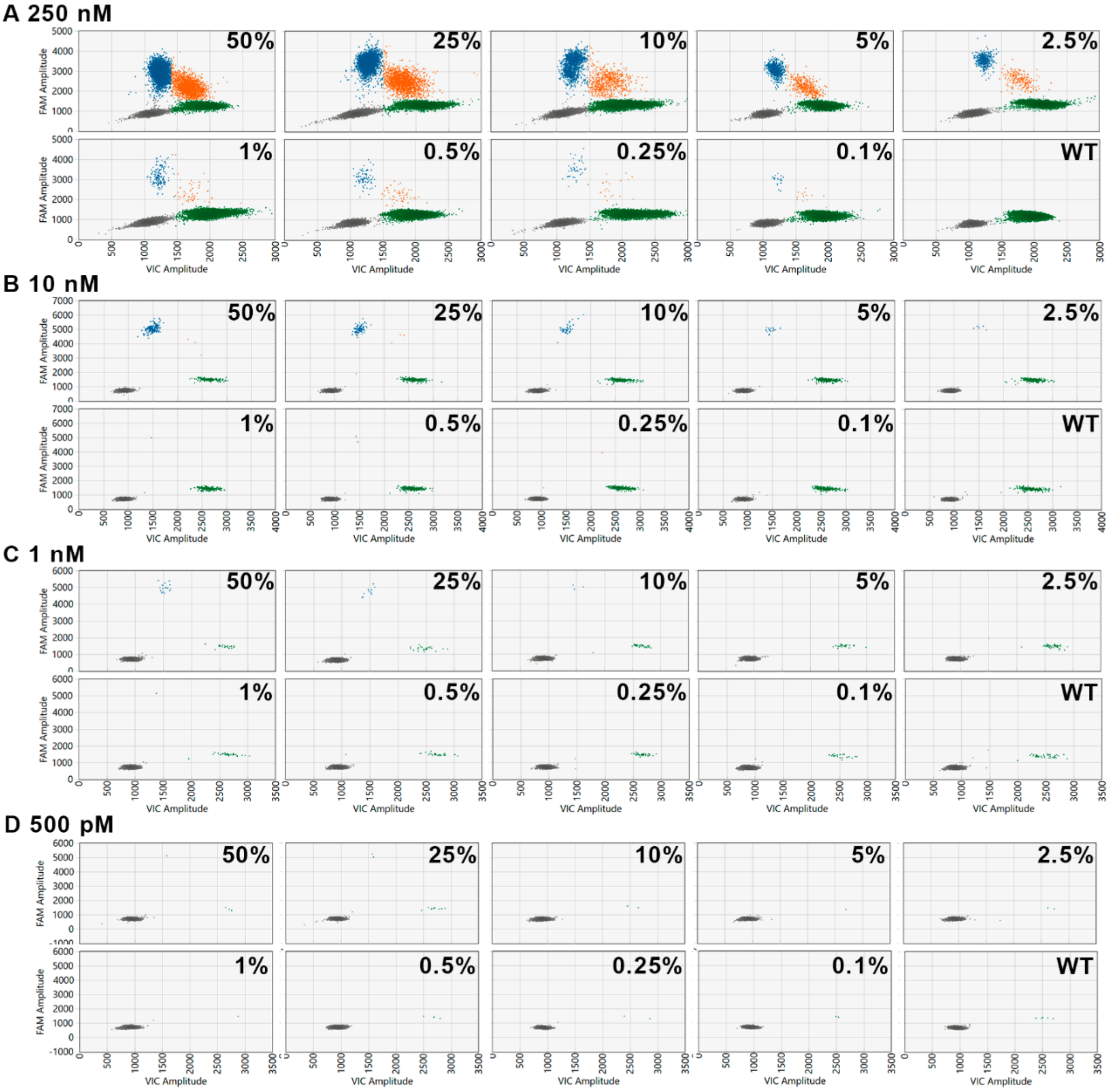

Supplementary Figure S5. ddPCR 2-D fluorescence amplitude plot of the BRAF VAF cohort at different DNA input concentrations.

VIC amplitude in the x axis represents the BRAF WT probe, and FAM amplitude in the y axis repesents the BRAF p.V600E probe. Alas, the black cluster on the plot represents the negative droplets, the green cluster represents the droplets that are positive for the BRAF WT alle only, the blue cluster represents the droplets that are positive for the BRAF p.V600E DNA only, and the orange cluster represents the droplets that are positive for both WT and mutant targets: **(A)** 250 nM, (**B)** 10nM, **(C)** 1 nM, and **(D)** 500 pM.

Supplementary Table S1: Conventional PCR primers.

Name	Sequence	Length	Tm	Amplicon size
**BRAF F**	5'- GAAATTAATACGACTCACTATAGGGTCATGAAG ACCTCACAGTAAAAATAGGTGATT -3'	57	64ºC	117
**BRAF R**	5'- ATTCTTACCATCCACAAAATGGATCCAGACAA -3'	32	59ºC	

Supplementary Table S2: crRNA sequences used in this study.

Name	Complete crRNA Sequence	Spacer sequence	Length
**BRAF wildtype**	5′- GAUUUAGACUACCCCAAAAACGAAGGGGACUAA AACUC**A**GUGUAGCUAGACCAAAAUCACCUAU-3′	5'- UC**A**GUGUAGCUAGA CCAAAAUCACCUAU -3′	84
**BRAF p.V600E**	5′- GAUUUAGACUACCCCAAAAACGAAGGGGACUAA AACUC**U**GUGUAGCUAGACCAAAAUCACCUAU-3′	5'- UC**U**GUGUAGCUAGA CCAAAAUCACCUAU -3′	84

Supplementary Table S3: Total copies of the target (RNA or DNA) for the different experimental concentrations (250, 10, and 1 nM) and allele frequencies (AF) (50, 25, 10, 5, 2.5, 1, 0.5, 0.1, and 0%) used.

**Target concentration**	**250 nM**		**10 nM**		**1 nM**	
**V600E AF (%)**	**WT (total copies)**	**V600E (total copies)**	**WT (total copies)**	**V600E (total copies)**	**WT (total copies)**	**V600E (total copies)**
**50**	8.0E+10	8.0E+10	3.2E+09	3.2E+09	3.20E+08	3.20E+08
**25**	1.2E+11	4.0E+10	4.8E+09	1.6E+09	4.8E+08	1.60E+08
**10**	1.4E+11	1.6E+10	5.8E+09	6.4E+08	5.76E+08	6.40E+07
**5**	1.5E+11	8.0E+09	6.1E+09	3.2E+08	6.08E+08	3.20E+07
**2.5**	1.6E+11	4.0E+09	6.2E+09	1.6E+08	6.24E+08	1.60E+07
**1**	1.6E+11	1.6E+09	6.3E+09	6.4E+07	6.34E+08	6.40E+06
**0.5**	1.6E+11	8.0E+08	6.4E+09	3.2E+07	6.37E+08	3.20E+06
**0.1**	1.6E+11	1.6E+08	6.4E+09	6.4E+06	6.39E+08	6.40E+05
**0**	1.6E+11	0.0E+00	6.4E+09	0.0E+00	6.40E+08	0.00E+00
